# Relationship Between Intraoperative Macroscopic Anterior Cruciate Ligament Findings, Perioperative Surgical Findings, and Postoperative Clinical Outcome After Cruciate-Retaining Total Knee Arthroplasty

**DOI:** 10.7759/cureus.54239

**Published:** 2024-02-15

**Authors:** Tsuneari Takahashi, Ryusuke Ae, Tatsuya Kubo, Yuya Kimura, Mikiko Handa, Katsushi Takeshita

**Affiliations:** 1 Department of Orthopaedic Surgery, Ishibashi General Hospital, Shimotsuke, JPN; 2 Division of Public Health, Center for Community Medicine, Jichi Medical University, Shimotsuke, JPN; 3 Department of Orthopaedic Surgery, Shin-Oyama City Hospital, Oyama, JPN; 4 Department of Orthopaedic Surgery, Nasu Chuo Hospital, Otawara, JPN; 5 Department of Orthopaedics, Jichi Medical University, Shimotsuke, JPN

**Keywords:** crp, knee osteoarthritis, c-reactive protein, knee stability, total knee arthroplasty, anterior cruciate ligament injury

## Abstract

Purpose: To assess how intraoperative macroscopical anterior cruciate ligament (ACL) findings affect perioperative procedures, biomarkers, and postoperative anterior-posterior (AP) laxity and range of motion (ROM) after cruciate-retaining (CR) total knee arthroplasty (TKA) and to determine how chronic ACL deficiency may affect postoperative inflammatory biomarker, AP laxity, and ROM.

Methods: A total of 121 patients with varus knee osteoarthritis without a history of ACL injury who underwent ATTUNE® (DePuy Synthes, Warsaw, IN) CR TKA were analyzed. Intraoperative ACL findings were stratified into intact, damaged, and diminished, according to the tension by probing, synovial coverage, and vascularity. C-reactive protein (CRP) levels were examined at one, seven, and 14 days after surgery. Knee AP laxity measurements using Kneelax 3 (Monitored Rehab Systems, Haarlem, The Netherlands) and postoperative knee ROM were also compared.

Results: One-way ANOVA showed significant differences in CRP levels examined one day after surgery observed between the three groups (8.4 (3.8), 9.8 (4.3), and 13.2 (7.7) mg/dL, respectively; P = 0.018), with post hoc analysis showing that CRP levels one day after surgery were significantly greater in the diminished group than in the intact and damaged groups (P = 0.012 and 0.023, respectively). AP laxity in 30° of knee flexion was observed between the three groups (5.4 (2.3), 5.8 (2.5), and 7.1 (2.8) mm, respectively; P = 0.039), with post hoc analysis showing that AP laxity in 30° of knee flexion was significantly greater in the diminished group than in the intact group (P = 0.038). Knee ROM showed no significant differences.

Conclusion: Intraoperative ACL diminishment was associated with higher CRP one day after surgery and midrange AP laxity one year after surgery.

## Introduction

Total knee arthroplasty (TKA) has been shown to alleviate pain and improve activities of daily living among patients with end-stage knee osteoarthritis [1]. However, up to 20% of patients are not satisfied after TKA [2,3]. Primary medial knee osteoarthritis in an anterior cruciate ligament (ACL)-intact knee usually involves the anteromedial aspect and is therefore called anteromedial osteoarthritis [4]. On the other hand, in cases of ACL deficiency, chronic instability typically results in a more extensive wear pattern that includes the posterior aspect of the medial compartment, a condition known as posterior medial osteoarthritis with stabilizing osteophytes [5,6].

C-reactive protein (CRP) elevation is a well-known laboratory finding after TKA, the levels of which usually decline to normal within three to six weeks after TKA, although some patient factors may affect this time period [7]. To the best of our knowledge, no studies on knee surgery have assessed whether intraoperative ACL findings affect postoperative inflammation and postoperative clinical outcomes such as anterior-posterior (AP) laxity and range of motion (ROM) after cruciate-retaining (CR) TKA.

In the current study, we hypothesized that intraoperative ACL findings (i.e., synovial coverage or preserved continuity and tension) could influence perioperative procedures, biomarkers, and postoperative clinical outcomes. After classifying intraoperative macroscopical ACL findings into three categories (intact, damaged, and diminished ACL), we aimed to determine how chronic ACL deficiency may affect postoperative inflammatory biomarkers, AP laxity, and ROM, and to assess the impact of TKA using ATTUNE® TKA (DePuy Synthes, Warsaw, IN) one year after surgery.

## Materials and methods

A total of 121 patients with knee osteoarthritis, without a history of ACL injury, who underwent ATTUNE® CR TKA from July 2018 to December 2021 were included in this retrospective study. An experienced knee surgeon determined the indication for TKA based on clinical findings (e.g., persistent knee pain despite conservative treatment, decreased activities of daily living, limping gait, and decreased ROM) and radiographic findings, according to the Kellgren and Lawrence classification grade 3 or 4 on standing knee radiographs [8]. Exclusion criteria were previous TKA, knee osteotomy, or ligament reconstruction due to the confounding effects of prior surgery on AP stability.

Surgical procedures

TKA was performed in all cases using a midvastus approach with a cemented implant of CR; ACL was dissected in all cases with the posterior cruciate ligament (PCL) retained. None of the patients underwent patellar resurfacing [9]. Mechanical alignment TKA was performed to obtain a mechanically neutral coronal mechanical limb alignment by cutting the femur and tibia so that the rectangular flexion and extension gaps were perpendicular to the mechanical axis. The distal femur was cut using an intramedullary alignment system. The posterior femur was cut using a reference guide to ensure that the osteotomy line was parallel to the surgical epicondyle line and perpendicular to the Whiteside's line [10]. The proximal tibia was cut using an extramedullary alignment system. Care should be taken not to damage the PCL during proximal tibial cutting. Also, care should be taken to create an adequate flexion gap with appropriate posterior tibial tilt. After insertion of the trial component, ROM and other intraoperative parameters of the knee were determined. To balance the gap between flexion and extension, release of the PCL, collateral ligament, and reticular ligament was not performed in all cases. Definitive components were introduced with cement fixation and a final evaluation of ROM was performed.

Measurements

Preoperative Patient Characteristics

Patient’s characteristics included age, body mass index (BMI), sex, affected side of knees, ROM for both extension and flexion of knees, and hip-knee angle (HKA; varus indicates plus). ROM was measured using a double-armed goniometer.

Intraoperative Evaluation of ACL

Intraoperative ACL findings were macroscopically evaluated by stratifying them into three groups: ACL with intact tension assessed by probing, well synovial coverage, and vascularity (intact group); loose ACL assessed by probing with decreased synovial coverage and vascularity (damaged group); and diminished ACL (diminished group).

Perioperative Findings

The medial and lateral osteotomy thicknesses of the distal femur, dorsal femur, and proximal tibia were measured using calipers. In addition, surgical time and C-reactive protein (CRP) levels were examined on postoperative days one, seven, and 14 as perioperative invasive indices [11].

Postoperative AP Laxity

**Figure 1:**
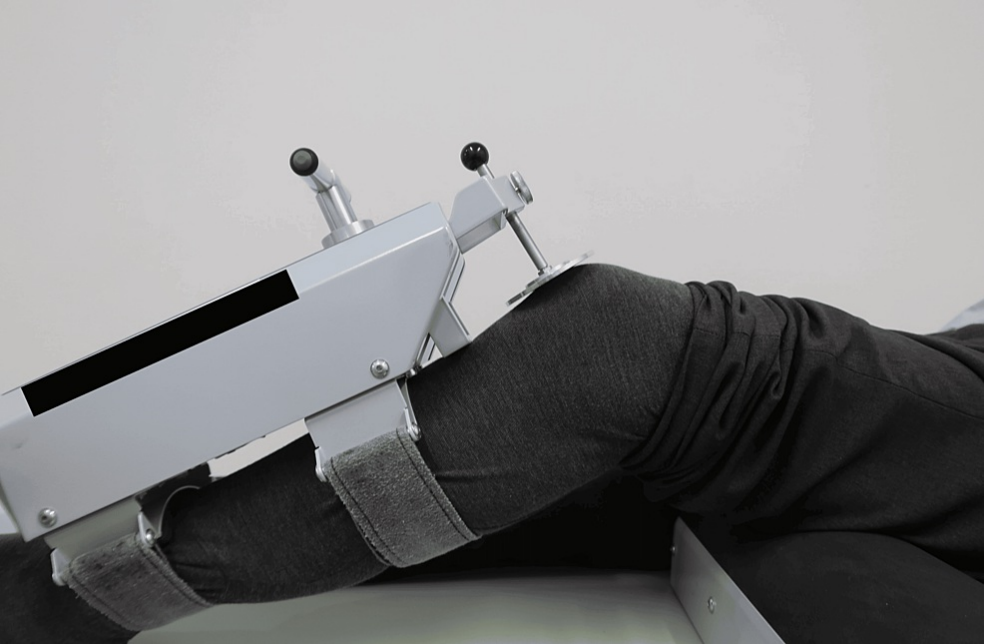
Postoperative knee anterior-posterior laxity measurements using the Kneelax tri-arthrometer.

AP laxity was assessed at regular follow-up visits one year after TKA. Each patient underwent knee AP laxity measurement using the Kneelax 3 (Monitored Rehab Systems, Haarlem, The Netherlands). This test was performed at 30° and 90° knee joint flexion upon awakening (Figure 1). One senior surgeon performed the AP drawer test and applied a force of 132 N to the tibia against the femur in the anteroposterior direction; the total AP laxity was calculated and recorded [9]. Additionally, ROM of knee extension and flexion was examined.

Statistical analysis

Numerical variables were presented as means and standard deviations (SDs), whereas categorical variables were presented as numbers and percentages. All statistical analyses were performed using EZR software [12]. For comparisons between three groups based on intraoperative ACL findings (intact, damaged, and diminished groups), one-way analysis of variance (ANOVA) for numerical variables and chi-square tests for categorical variables were employed. Furthermore, post hoc analyses using Bonferroni correction were performed to assess differences between two groups (intact vs. damaged groups; intact vs. diminished groups; and damaged vs. diminished groups) when significant differences were found in initial comparison analyses for the three groups. The significance threshold for statistical tests was set at P < 0.05.

To avoid introducing beta errors into the present results, sample size calculation was performed in advance to estimate the minimum number of patients for this study, which resulted in 66 patients (α error of 0.05, β error of 0.20, and Cohen’s effect size of 0.4, calculated using G*Power 3.1, Franz Paul, Kiel, Germany) [13]. Based on this calculation, we confirmed that the sample size of the study (N = 121) was sufficient to determine the statistical significance.

## Results

Of the 121 patients who underwent TKA, 40, 58, and 23 patients were classified into intact, damaged, and diminished groups, respectively, based on intraoperative ACL evaluation. Comparison of patient characteristics (Table 1) revealed no significant differences among the three groups with respect to age, BMI, male-to-female ratio, and affected side of the knee.

**Table 1:** Comparison of preoperative patient characteristics, perioperative findings, and postoperative anterior-posterior laxity according to the intraoperative findings of anterior cruciate ligament. Numerical data are presented as mean (standard deviation). * Presented as the number (percentage) of patients. P < 0.05 is considered statistically significant. ^†^ Chi-square tests for sex and affected side; one-way analysis of variance (ANOVA) for other numerical variables. ^‡^ Assessments one year after surgery. ACL: anterior cruciate ligament; SD: standard deviation; BMI: body mass index; ROM: range of motion; HKA: hip-knee angle; CRP: C-reactive protein.

Variables	Intraoperative ACL findings			P-value^†^
	Intact (n = 40)	Damaged (n = 58)	Diminished (n = 23)	
	Mean (SD)	Mean (SD)	Mean (SD)	
Preoperative patient characteristics				
Age (years)	72.1 (7.8)	72.3 (7.5)	72.6 (7.5)	0.96
BMI (kg/m^2^)	25.9 (3.8)	25.7 (4.0)	25.6 (3.9)	0.94
Sex (male)^*^	11 (27.5%)	11 (19.0%)	6 (26.1%)	0.76
Affected side (right)^*^	21 (52.5%)	35 (60.3%)	9 (39.1%)	0.29
Preoperative ROM for extension (°)	−7.1 (6.9)	−10.3 (7.4)	−9.8 (6.5)	0.08
Preoperative ROM for flexion (°)	124.0 (12.1)	118.1 (12.5)	118.9 (13.2)	0.07
Preoperative HKA (°)	10.7 (5.9)	11.2 (7.0)	17.3 (5.4)	<0.001
Perioperative findings: thickness of bony resection				
Distal femur (mm)				
Medial/lateral	7.2 (1.3)/6.8 (1.5)	7.5 (1.3)/7.0 (1.3)	6.8 (1.6)/7.0 (1.3)	0.13/0.64
Dorsal femur (mm)				
Medial/lateral	9.4 (2.0)/7.0 (1.7)	9.4 (1.7)/7.0 (1.4)	9.8 (2.0)/7.4 (1.8)	0.57/0.63
Proximal tibia (mm)				
Medial/lateral	4.2 (1.8)/10.5 (1.3)	4.5 (2.2)/10.5 (1.5)	2.4 (2.0)/11.5 (2.3)	<0.001/0.028
Surgical time (minutes)	100.2 (18.8)	97.0 (10.7)	101.0 (16.5)	0.43
CRP examined after surgery (mg/dL)				
1 day	8.4 (3.8)	9.8 (4.3)	13.2 (7.7)	0.0018
7 days	4.0 (3.4)	3.7 (2.3)	4.0 (3.2)	0.88
14 days	0.80 (0.95)	0.98 (1.1)	1.0 (1.4)	0.67
Postoperative anterior-posterior laxity^‡^				
30° of knee flexion (mm)	5.4 (2.3)	5.8 (2.5)	7.1 (2.8)	0.039
90° of knee flexion (mm)	2.6 (2.0)	2.5 (1.7)	3.1 (1.9)	0.38
ROM for extension (°)	−1.4 (2.8)	−1.6 (3.8)	−0.87 (2.5)	0.63
ROM for flexion (°)	120.1 (11.0)	117.0 (11.9)	117.2 (10.6)	0.38

**Figure 2:**
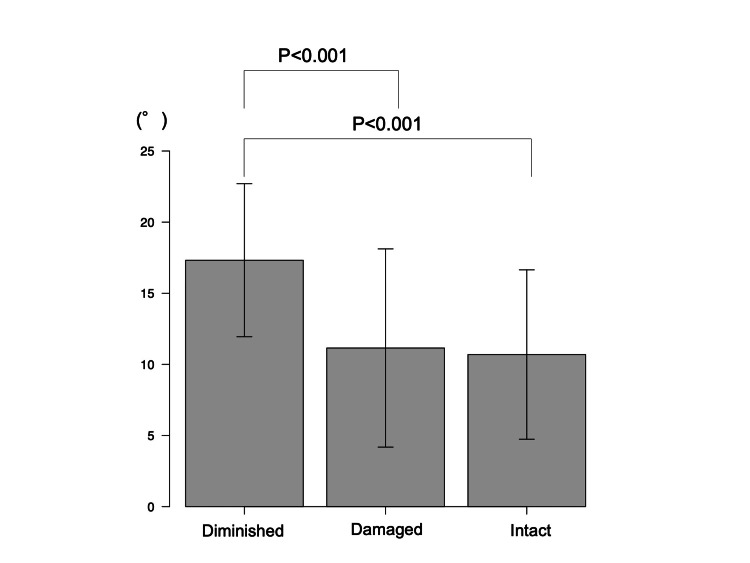
Post hoc analysis of preoperative hip-knee angle. P < 0.05 is considered statistically significant.

On the other hand, one-way ANOVA showed significant differences in preoperative HKA (mean (SD): 10.7° (6.0°) in the intact group, 11.2° (7.0°) in the damaged group, and 17.3° (5.4°) in the diminished group; P < 0.001). Post hoc analysis showed that preoperative HKA in the diminished group was significantly greater than in the intact and damaged groups (P < 0.001 and < 0.001, respectively) (Figure 2).

**Figure 3:**
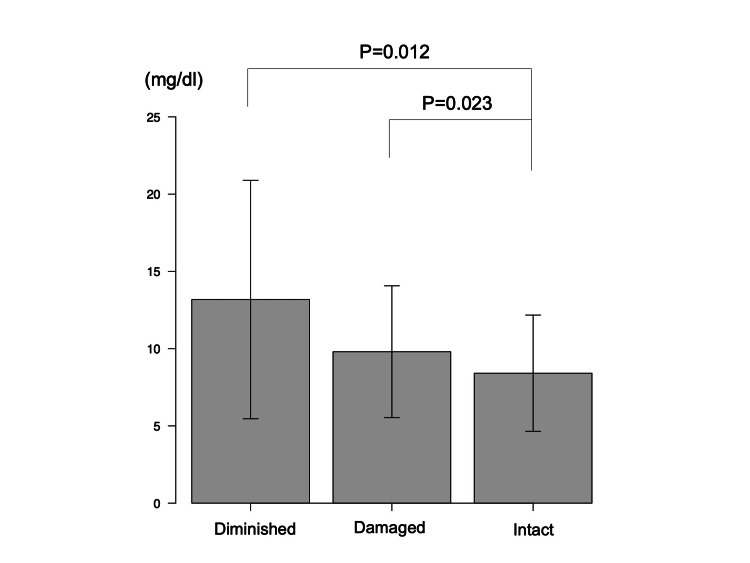
Post hoc analysis of C-reactive protein on postoperative day one. P < 0.05 is considered statistically significant.

A comparison of perioperative findings (Table 1) revealed no significant differences in distal femoral or dorsal osteotomy thickness. On the other hand, there was a significant difference in the medial osteotomy thickness of the proximal tibia (4.2 (1.8), 4.5 (2.2), and 2.4 (2.0) mm, respectively; P < 0.01). Post hoc analysis showed that the medial osteotomy thickness of the proximal tibia was significantly greater in the intact and damaged groups than in the diminished group (P < 0.001 and P < 0.001, respectively). In addition, significant differences in the bony resection thickness on the lateral side of the proximal tibia were found (10.5 (1.3), 10.5 (1.5), and 11.5 (2.3) mm, respectively; P = 0.028). Post hoc analysis showed that the bony resection thickness on the lateral side of the proximal tibia in the intact and damaged groups was significantly greater than that in the diminished group (P = 0.044 and 0.042, respectively). Furthermore, there was a significant difference in CRP levels tested on postoperative day one between the three groups (8.4 (3.8), 9.8 (4.3), and 13.2 (7.7) mg/dL, respectively; P = 0.018), and post hoc analysis showed that CRP levels on postoperative day one were significantly greater in the intact and damaged groups than in the diminished group (P = 0.012 and 0.023, respectively) (Figure 3).

CRP values on postoperative days seven and 14 were not significantly different among the three groups.

**Figure 4:**
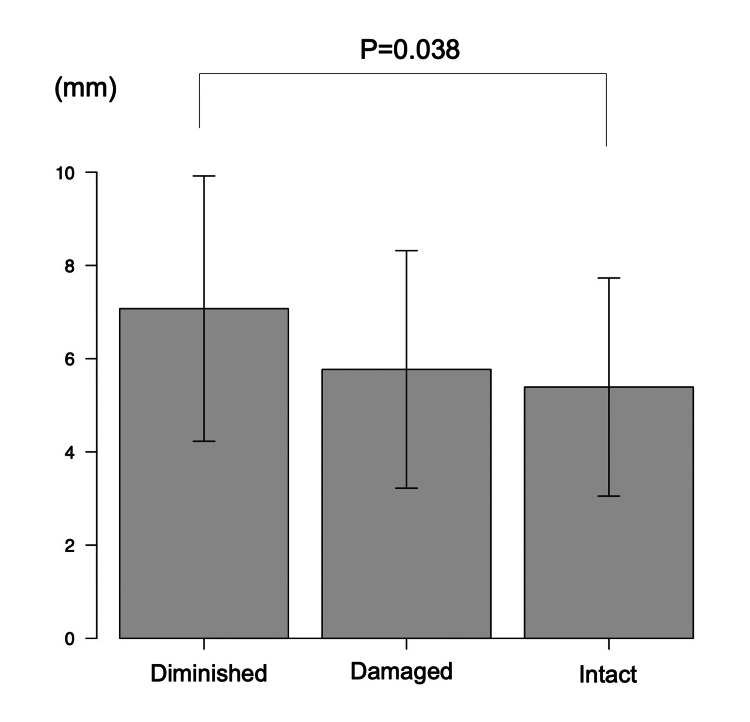
Post hoc analysis of anterior-posterior laxity measurements using Kneelax 3 arthrometer in 30° of knee flexion. P < 0.05 is considered statistically significant.

AP laxity assessment one year after TKA (Table 1) showed no significant differences in 90° of knee flexion. On the other hand, significant differences in AP laxity in 30° of knee flexion were observed between the three groups (5.4 (2.3), 5.8 (2.5), and 7.1 (2.8) mm, respectively; P = 0.039), with post hoc analysis showing that AP laxity in 30° of knee flexion was significantly greater in the diminished group than in the intact group (P = 0.038) (Figure 4).

ROM in knee extension and flexion were not significantly different among the three groups (Table 1).

## Discussion

The most important findings of the current study were that intraoperative ACL findings affected perioperative invasive findings and midrange AP laxity one year after surgery. Accordingly, our findings showed that diminished intraoperative ACL was associated with greater preoperative varus deformity, resulting in a larger lateral tibial and smaller medial tibial bony resection thickness upon surgery with higher CRP one day after surgery. Notably, intraoperative ACL findings did not significantly influence postoperative knee ROM for both extension and flexion one year after surgery. On the other hand, intraoperative ACL findings significantly influenced postoperative AP laxity of the knee at 30°, indicating that the influence of intraoperative diminished ACL remained as midrange AP laxity after CR TKA without soft tissue release even one year after surgery.

Varus deformity is more likely to occur in ACL-deficient knees [14]. Patients with ACL deficiency typically exhibit more extensive wear patterns, which can be a plausible reason for the larger preoperative HKA and larger proximal tibial bony resection thickness upon surgery. Demey et al. [15], who compared patients with and without previous history of ACL injury who underwent TKA, concluded that postoperative clinical outcomes were comparable between the groups, a finding partially consistent with our results. However, we excluded patients with a history of ACL injury and compared intraoperative ACL findings, indicating hidden ACL deficiency - a novelty of our study.

Our findings showed that CRP levels one day after surgery were significantly higher in patients with diminished ACL than in those with intact and decreased ACL findings, indicating that patients with diminished ACL may have undergone the most invasive surgery. As such, chronic ACL deficiency might induce more postoperative inflammation. In the future, we are interested in determining whether the anti-inflammatory effects of dexamethasone can better attenuate the acute phase reaction in patients with diminished ACL [16].

Postoperative AP laxity in 30° of knee flexion was significantly larger in diminished ACL cases. Mullis et al. [6] described that chronic ACL-deficient knees had stabilizing osteophytes at the posterior lateral corner of the medial tibial plateau resected at the time of TKA surgery, which allowed those knees to adapt to improve stability. Our results indicated that chronic ACL deficiency did influence the postoperative midrange AP laxity.

These results indicate that CRP one day after surgery and midrange AP laxity were significantly higher in the ACL diminished group, even though ROM was comparable one year postoperatively. This helps surgeons consider implant selection and postoperative anti-inflammatory therapy in knee osteoarthritis patients with chronic ACL deficiency.

Limitations

The present study has several limitations that are worth mentioning. First, intraoperative ACL findings were subjective. Second, AP laxity was measured rigorously at a force of 132 N, but interobserver variability in instrumented measurements was not assessed. Third, all TKA procedures were CR TKAs performed by a single consultant knee surgeon using a midvastus approach. Therefore, these results cannot be generalized to TKA using other approaches or to cruciate arthroplasty or posterior stabilized TKA. Finally, a comparison of preoperative and postoperative joint lines, which may affect AP laxity, was not performed in this study. However, this is a topic for future research.

Despite these limitations, this is the first study to show that AP laxity at one year postoperatively is associated with intraoperative ACL findings, suggesting that ACL deficient patients may have similar postoperative AP laxity as intact ACL cases after TKA with GRADIUS™ design implants. Future comparative studies are needed to determine if these findings translate into longer implant survival.

## Conclusions

Intraoperative ACL diminishment was associated with greater preoperative varus deformity and resulted in larger lateral and smaller medial tibial bony resection thickness at the time of surgery. Furthermore, intraoperative ACL diminishment resulted in higher CRP one day after surgery and midrange AP laxity one year after surgery. However, the postoperative knee ROM one year after surgery did not significantly differ regardless of intraoperative ACL findings.
